# Cataract surgery outcome in patients with non-glaucomatous pseudoexfoliation

**DOI:** 10.22336/rjo.2017.36

**Published:** 2017

**Authors:** Praveen Venkatesha Sastry, Anuj Kumar Singal

**Affiliations:** * Department of Ophthalmology, JSS Hospital, Mysore, India

**Keywords:** corneal edema, exfoliation, intraocular pressure, glaucoma, visual acuity

## Abstract

**Aim.** To study the outcome of cataract surgery in eyes with pseudoexfoliation without signs of glaucoma.

**Methods.** This prospective study was done over nine months on patients with pseudoexfoliation undergoing small incision cataract surgery. Only patients with normal intraocular pressure (Central Corneal Thickness Corrected), normal cup disc ratio and open angles were included.

Patients on anti-glaucoma medication were excluded. Patients underwent surgery performed by a single senior surgeon. Intraoperative and postoperative day (POD) 1 findings were noted.

**Results.** The mean age of the patients was 61.60 years +/ - 10.21 years. Males were 46% (n=16). Right eye was operated upon in 60% of the cases (n=21). The mean pupil dilation was 5.1 mm +/ - 1.47 mm. The postoperative day 1 visual acuity of the patients was logMar 1.02 +/- 0.64 and the mean POD 1 intraocular pressure was noted to be 26.23 mmHg +/ - 11.40 mmHg.

Five cases had a zonular dialysis intraoperatively due to weak zonules. 11 cases had high anterior chamber reaction of 2+ or more. Four cases had unavoidable sphincter tears and two cases had iridodialysis superiorly during nucleus delivery.

**Conclusions.** Intraoperative complications should be anticipated in patients with pseudoexfoliation even without glaucomatous changes due to poor pupillary dilation and zonular weakness. First postoperative day visual acuity in pseudoexfoliation patients undergoing cataract surgery was found to be low due to severe anterior chamber inflammation causing elevated intraocular pressure and corneal edema.

**Abbreviations.** POD = Postoperative day, PEX = Pseudoexfoliation syndrome, LOXL 1 = Lysyl oxidase-like 1, IOP = Intraocular pressure, AC = Anterior chamber, IOL = Intraocular lens

## Introduction

The Pseudoexfoliation syndrome (PEX) was first described by Lindberg in 1917 in Finnish patients. The histological studies done by Davork-Theobald differentiated between the exfoliation seen in glass blowers and senile exfoliation. Senile exfoliation was then termed as Pseudoexfoliation [**1**].

Pseudoexfoliation is characterized by production and deposition of abnormal fibrillar extracellular material in the ocular adnexa, Iris and Lens Capsule and has also been observed in other tissues such as blood vessels, skin, gallbladder, kidneys, lungs, and heart. It has been linked to dysregulation of lysyl oxidase-like 1 (LOXL1) gene in PEX tissues [**2**].

Nordic countries tend to have a higher rate of pseudoexfoliation with up to 22.4% in Finland, above 60 years of age [**3**,**4**]. Rates in most other populations around the world are less but have been found to be as high as in American Navajo Indians (38.0%), to the very low rate in ethnic Chinese of Singapore (0.7%) [**5**,**6**]. 

Due to its manifestations, pseudoexfoliation can have a deleterious effect intraoperatively and significant effects on visual prognosis, postoperatively. Pseudoexfoliation Patients undergoing cataract surgery have been associated with various intraoperative cataract surgery complications such as zonular weakness, non-dilating pupil, Iris atrophy, and corneal edema [**7**]. Studies of unilateral exfoliation syndrome have found about a 5% decrease in the number of endothelial cells in the affected eye and about a 14% decrease in endothelial counts when compared to normal eyes [**8**-**10**]. Mild increase in polymegathism and pleomorphism of endothelial cells has also been noted [**8**,**9**]. 

PEX is the commonest identifiable cause of secondary glaucoma and most of these complications are thought to be secondary to glaucoma and associated to raised intraocular pressure rather than pseudoexfoliation itself [**7**].

In this first of a kind study, the effect of pseudoexfoliation without glaucomatous changes on the outcome of cataract surgeries has been studied. We aimed to find a correlation between postoperative visual acuity and pseudoexfoliation and try to understand the ocular manifestations of pseudoexfoliation and its resulting clinical and surgical complications.

## Material and Methods

The study was done over nine months in a tertiary hospital in south India. Over 900 patients attended for cataract surgery during this period but only 35 met the inclusion and exclusion criteria and were taken for the study. All the patients with pseudoexfoliative material seen on slit lamp examination on Iris/ Corneal endothelium/ pupillary ruff/ Lens Capsule along with lens opacities were included but if associated glaucomatous changes such as raised intraocular pressure and Glaucomatous Optic Disc were found, those patients were rejected. All the patients with history of secondary glaucoma, post refractive surgery were also excluded and so were the patients with obvious zonular weakness and phacodonesis or iridodonesis. A written informed consent was obtained for participation in the study from all the cases, according to Helsinki Declaration of 1975, as revised in 2000 and 2008.

A complete ophthalmic evaluation was done, including the recording of relevant ocular history; best corrected visual acuity with Snellen’s chart, examination of the pupillary reaction, and slit lamp evaluation of the anterior segment with a careful search for pseudoexfoliative deposits. Intraocular pressure (IOP) was recorded by Goldman’s applanation tonometry along with Gonioscopy by using a Goniolens in complete sterile condition in sitting position by a single fellow (blinded for patient details). Only subjects with open angles were included. Lens changes were graded by using Lens Opacities Classification System III (LOCS III) system and presence of subluxation of lens/ Phacodonesis was noted.

For easy reference, authors used a clinical grading system for classification, which was the following:

M1-MILD-Those with Pseudoexfoliative deposits on pupillary margin and anterior capsule.

M2-MODERATE-Those with M1 Iris changes (such as Iris atrophy).

M3-SEVERE-Those with M1/ M2 + Pseudoexfoliative deposits on endothelium and angle or other anterior segment structures.

Detailed stereoscopic evaluation of the fundus and the optic disc with the indirect ophthalmoscope and the +90 D lens was performed. Patients with dense cataracts, with no fundus view, underwent fundus examination postoperatively and if found to have a glaucomatous optic disc then were excluded from the study.

Glaucomatous optic nerve damage was considered as one or more of the following features:

(i) Vertical cup-disc ratio of 0.5 or more (physiological cups were excluded);

(ii) Vertical cup-disc asymmetry of 0.2 or more between the two eyes;

(iii) Characteristic glaucomatous excavation of the neuroretinal rim.

Cataract surgery by phacoemulsification was performed for all the patients by a single senior consultant trained in the surgery. All the patients were prescribed topical ketorolac 0.3% for one day preoperatively to decrease the intraoperative miosis. Topical Tropicacyl Plus eye drops (Tropicamide 0.8% w/ v+ Phenylephrine 5% w/ v) was used the same morning to achieve mydriasis. Maximum pupil dilation pre-operative was noted. 

During surgery, intracameral adrenaline along with copious amounts of viscoelastic material was used to achieve mydriasis in cases with inadequate mydriasis. Few cases required Iris hooks (8 mm* 0.8mm BD Visitec™ micro iris hooks) to achieve sufficient mydriasis. Phacoemulsification within the bag placement of foldable intraocular lens (Hydrophobic Acrylic Sensar® IOL) was done. In five cases an in the bag placement was not possible due to zonular dialysis and thus the lens was placed in the sulcus in three cases, whereas scleral fixation of the lens was done in the remaining two cases.

Intraoperative and first day postoperative complications along with best-corrected visual acuity were noted. IOP measurement was done on postoperative day 1 by Goldmann Applanation tonometry in complete sterile condition in sitting position by a single fellow (blinded for patient details). A detailed assessment of the anterior segment including the anterior chamber reaction, corneal edema, IOL position, iris tissue, as well as detailed fundus examination was done. Statistical tools used were regression analysis and paired t test. A P value less than or equal to 0.05 was considered significant. Microsoft Excel 2013 was used for data collection, tabulation, and chart preparation.

## Results

The prospective study was done over nine months, in which 35 patients with pseudoexfoliation underwent phacoemulsification by a single surgeon trained in the same. The mean age of the patients was 61.60 years +/ - 10.21 years with the maximum number of patients being in the 61-70 years age group (n=14) as depicted in **Table 1**. 

**Table 1:** Age groups of patients

AGE GROUPS	No. of Patients (%)
31-40	2 (5.71%)
41-50	3 (8.57%)
51-60	11 (31.43%)
61-70	14 (40%)
71-80	4 (11.43%)
81-90	1 (2.86%)

Males represented 46% (n=16). Right eye was operated upon in 60% of the cases (n=21). 19 cases (54.29%) were immature cataract while 16 (45.71%) were mature cataract. Different types of cataract grading are depicted in **Table 2**.

**Table 2:** Grades of cataract

Cataract Grading	No. of Patients (%)
Grade II Nuclear Sclerosis	6 (17.14%)
Grade III Nuclear Sclerosis	4 (11.43%)
Grade IV Nuclear Sclerosis	6 (17.14%)
Mature cataract	11 (31.43%)
Hypermature cataract	5 (14.29%)
Intumescent cataract	1 (2.86%)
Posterior subcapsular cataract	2 (5.71%)

Eighteen patients (51.43%) had mild pseudoexfoliation (M1), while five (14.29%) had moderate (M2) and twelve patients (34.29%) had severe pseudoexfoliation (M3).

**Fig. 1a:**
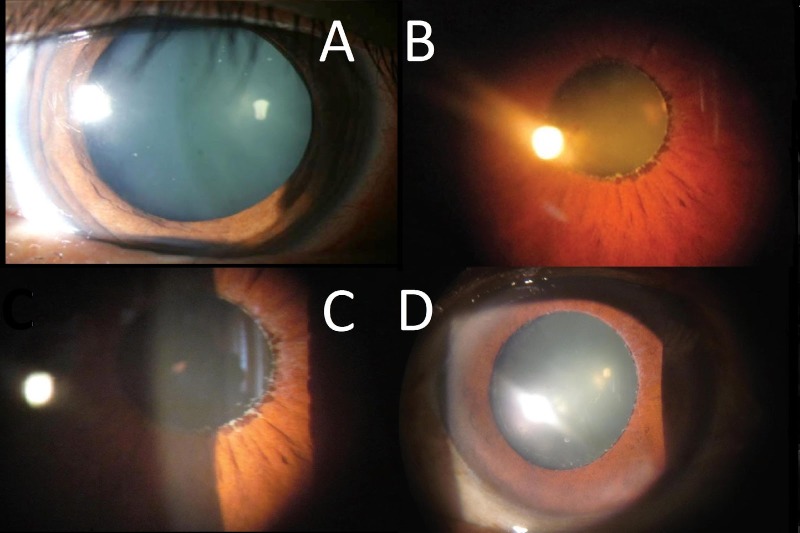
Normal Pupil dilation, **Fig. 1b** Poor Mydriasis on the day of surgery, **Fig. 1c** Moderate Mydriasis on the day of surgery with pseudoexfoliative material visible on pupillary margin, **Fig. 1d** Good Mydriasis on the day of surgery

**Fig. 1** demonstrates the different amounts of pupil dilation observed. The mean pupil dilation was 5.1 mm +/ - 1.47 mm with 86% of the patients (n=30) having a maximum dilation of less than 6 mm as depicted in **Table 3**.

**Table 3:** Preoperative pupil dilation

Pupil Dilation	No. of patients
Poor (<4 mm)	14 (40%)
Moderate (5-6 mm)	16 (45.71%)
Good (>6 mm)	5 (14.29%)

The postoperative day 1 visual acuity of the patients was logMar 1.02 +/ - 0.64 roughly being equivalent to 20/ 210 on Snellen’s chart. The mean postoperative day 1 intraocular pressure was noted to be 26.23 mmHg +/ - 11.40 mmHg with ten patients (28.57%) having more than 35 mmHg as depicted in **Table 4** and required antiglaucoma medications.

**Table 4:** Postoperative day-1 intraocular pressure

Postoperative Day-1 Intraocular Pressure (mmHg)	No. of Patients
<20	11 (31.43%)
21-25	11 (31.43%)
25-30	1 (2.86%)
30-35	2 (5.71%)
35-40	4 (11.43%)
>40	6 (17.14%)

The postoperative day 1 visual acuity was found to be directly associated with the severity of pseudoexfoliation (p=0.001), Anterior chamber (AC) reaction (p<0.001), postoperative day 1 IOP (p=0.005) and pupil dilation (p=0.01), whereas no relationship was found with the type and density of cataract (p = 0.985).

Furthermore, postoperative IOP was itself found to be directly associated with the severity of pseudoexfoliation (p = 0.05) and anterior chamber reaction (p = 0.032). Corneal edema was also found to be more in patients with increasing levels of pseudoexfoliation (p = 0.026) and so was the anterior chamber reaction, which was found to be higher with higher grades of pseudoexfoliation (p=0.038).

Five cases had a zonular dialysis intraoperatively due to weak zonules noticed on the table. Two cases required scleral fixation, while in the other three, the lens was kept in the sulcus, while in the remaining 30 cases, foldable intraocular lens was placed in the bag. 11 cases had an AC reaction of 2+ or more, while four cases had unavoidable sphincter tears and two cases had a superior iridodialysis due to poor mydriasis as depicted in **Table 5**.

**Table 5:** Intraoperative complications

Complications	No. of Patients
Iridodialysis	2 (5.71%)
Zonular Dialysis	5 (14.29%)
Scleral Fixated Intraocular lens	3 (8.57%)
Sulcus Implantation	2 (5.71%)
Sphincter tears	4 (11.43%)
Exudative membrane	2 (5.71%)

## Discussion

PEX syndrome is an age as well as genetically determined generalized fibrotic matrix process of worldwide significance. It not only causes severe chronic open-angle glaucoma but also a spectrum of other serious spontaneous and surgical intraocular complications. Previous epidemiological studies of PEX have shown that it is more common in patients older than 60 years and prevalence that further increases with age, similar to the mean age of the patients (61.60 years) in our study [**10**]. Reports regarding sex predilection in PEX are conflicting. Some previous studies have shown male preponderance, while in 2003, Aravind et al. showed no sex predilection [**11**]. Avramides, Sakkias and Traindis reported a female preponderance [**12**]. Our study also showed no sex predilection as males constituted 46% of the cases.

In their study on 6046 patients, French et al. found that over 50% of the patients with pseudoexfoliation suffer from pseudoexfoliative glaucoma [**13**]. Such patients have higher chances of intraoperative as well as postoperative complications and thus were excluded from our study to determine the pure effect of pseudoexfoliation on cataract surgery. 

Ideally, a complete mydriasis of eight to nine mm is required for a good cataract surgery, however, none of the patients achieved the same and, in fact, most of the patients (60%, n=21) had a maximum dilation of less than six mm in spite of the use of mydriatics, as well as well as Nonsteroidal anti-inflammatory drugs (nsaids) like Ketorolac 0.3%. 

Previous studies, like that by Kuchle M et al. [**14**], of 436 patients, have found a statistically significant increase in intraoperative and postoperative complications in cataract extraction in eyes with pseudoexfoliation syndrome. The mean IOP on POD1 was found to be 26.23 mmHg (+/ - 11.40 mmHg), which is quite higher than 19.3±7.1mmHg as observed by Kim et al. [**15**] and a 4.6 mmHg increase from the study by Rainer et al. done in a normal population [**16**]. Furthermore, we found that 12 patients had IOP of more than 30 mmHg and six had more than 40 mmHg on postoperative day 1, requiring administration of hyperosmotic agents such as glycerol syrup.

**Fig. 2a:**
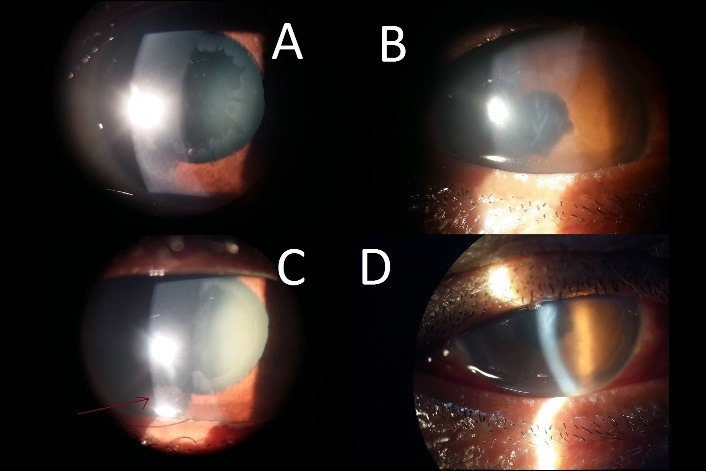
Preoperative eye with pseudoexfoliation seen on anterior capsule, **Fig. 2b **Postoperative same eye showing corneal edema and sphincter tear, **Fig. 2c** Preoperative eye with pseudoexfoliation on anterior lens capsule and corneal endothelium marked with arrow, **Fig. 2d **Postoperative day 1 of eye depicted in **1c** showing Lid edema, corneal edema and increased AC reaction

The postoperative day 1 vision was found to be logMar 1.02 (+/ - 0.64) or 20/ 210 on Snellen’s visual acuity chart, which is much less than the expected postoperative day 1 vision of 20/ 40 in non pseudoexfoliative eyes in today’s era [**17**]. Furthermore, the AC reaction was also found to be associated with postoperative IOP (p=0.032) and pseudoexfoliation (p=0.038). In their study, Shastri et al. [**18**] also observed an increased flare postoperatively, similar to our study, in which 11 cases (31.42%) had a flare of 2+ or more. Corneal edema, which is a common cause of decreased postoperative vision, was also found to be directly associated with pseudoexfoliation (p=0.026), as shown in **Fig. 2**.

Scorolli et al. [**19**] did an extensive research on 1052 patients and found that patients with PEX have five times a greater risk of intraoperative complications in cataract surgery compared to normal cases. He further stated that the recognition of this condition is very important before starting surgery on such patients. In our study, five cases had zonular dialysis requiring Intraocular lens (IOL) to be placed in the sulcus in two cases and scleral fixated IOL to be implanted in three cases and was possible due to high anticipation of complications in such cases. Furthermore, superior iridodialysis occurred in two cases and four cases had unavoidable sphincter tears due to small pupils. 

In this improved surgical expertise era, all the patients expect a good postoperative result and demand an excellent vision on the first postoperative day itself. Intraoperative complications should be anticipated in patients with pseudoexfoliation even without glaucomatous changes probably due to poor pupil dilation and zonular weakness. Furthermore, these patients usually have a higher postoperative AC reaction attributed to the increased handling of tissues and pupil stretching leading to decreased POD 1 vision. In our study, increased intraocular pressure and corneal edema on the first postoperative day caused a further decrease in the first postoperative day visual acuity. Thus, we recommend that all the patients with pseudoexfoliation should be offered a detailed counseling about cataract surgery with its possible outcomes and warn them regarding an adverse outcome of resultant visual acuity on the first postoperative day, which may or may not improve.

This study is the first one that relates pseudoexfoliation with the outcome of cataract surgery without the component of preoperative glaucoma and increased intraocular pressure. Future studies involving a larger population size are needed to have a better outlook on pseudoexfoliation and its effect on cataract surgery, as the small sample size was the limitation of this study.

**Source(s) of support**

Nil

**Conflicting Interest **

Nil

**Acknowledgement **

Dr. Parijat Joshi for relentless proofing of the manuscript. Mr. Ankush Singhal for his useful inputs in the statistical analysis.

## References

[1] Lindberg JGMD (1917). Clinical studies of depigmentation of the pupillary margin and transillumination of the iris in cases of senile cataract and also in normal eyes of the aged. Thesis.

[2] Schlötzer-Schrehardt U, Pasutto F, Sommer P, Hornstra I, Kruse FE, Naumann GOH, Zenkel M (2008). Genotype-Correlated Expression of Lysyl Oxidase-Like 1 in Ocular Tissues of Patients with Pseudoexfoliation Syndrome/Glaucoma and Normal Patients. The American Journal of Pathology.

[3] Krause U, Alanko HI, Karna J, Miettinen R, Larmi T, Jaanio E, Ollila OI, Takala J (1988). Prevalence of exfoliation syndrome in Finland. Acta Ophthalmol.

[4] Backhaus B, Lorentzen SE (1966). Prevalence of pseudoexfoliation in non glaucomatous eyes in Denmark. Acta Ophthalmol.

[5] Faulkner HW (1971). Pseudo exfoliation of the lens among the Navajo Indians. Am J Ophthalmol.

[6] Foster PJ, Seah SK (2005). The prevalence of pseudoexfoliation syndrome in Chinese people: the Tanjong Pagar Survey. Br J Ophthalmol.

[7] Repo LP, Teräsvirta  ME, Tuovinen EJ (1990). Generalized peripheral iris transluminance in the pseudoexfoliation syndrome. Ophthalmology.

[8] Vannas A, Setälä  K, Ruusuvaara P (1977). Endothelial cells in capsular glaucoma. Acta Ophthalmol.

[9] Miyake K, Matsuda  M, Inaba M (1989). Corneal endothelial changes in pseudoexfoliation syndrome. Am J Ophthalmol.

[10] Inoue K, Okugawa  K, Oshika T, Amano S (2003). Morphological study of corneal endothelium and corneal thickness in pseudoexfoliation syndrome. Eur J Ophthalmol.

[11] Sekeroglu MA, Bozkurt  B, Irkec M, Ustunel S, Orhan M, Saracbasi O (2008). Systemic associations and prevalence of exfoliation syndrome in patients scheduled for cataract surgery. Eur JOphthalmol.

[12] Avramides S, Traianidis  P, Sakkias G (1997). Cataract surgery and lens implantation in eyes with exfoliation syndrome. J Cataract Refract Surg.

[13] French DD, Margo  CE, Harman LE (2012). Ocular Pseudoexfoliation and Cardiovascular Disease: A National Cross-Section Comparison Study. North American Journal of Medical Sciences.

[14] Küchle M, Schönherr  U, Dieckmann U (1989). Risk factors for capsular rupture and vitreous loss in extracapsular cataract extraction. The Erlangen Ophthalmology Group. Fortschr Ophthalmol.

[15] Kim JY, Jo  MW, Brauner SC, Ferrufino-Ponce Z, Ali  R, Cremers SL, An Henderson B (2011). Increased intraocular pressure on the first postoperative day following resident-performed cataract surgery. Eye.

[16] Rainer G, Menapace  R, Findl O, Kiss B, Petternel V, Georgopoulos  M (2001). Intraocular pressure rise after small incision cataract surgery: a randomized intraindividual comparison of two dispersive viscoelastic agents. Br J Ophthalmol.

[17] Thanigasalam T, Reddy  SC, Zaki RA (2015). Factors associated with complications and postoperative visual outcomes of cataract surgery; A study of 1,632 cases. J Ophthalmic Vis Res.

[18] Shastri L, Vasavada  A (2001). Phacoemulsification in Indian eyes with pseudoexfoliation. J Cataract Refract Surg.

[19] Scorolli D, Campos  EC, Bassein L, Meduri RA (1998). Pseudoexfoliation syndrome. A cohort study on intraoperative complications in cataract surgery. Ophthalmologica.

